# Assessment of nursing staff attitude regarding prevention of cervical cancer

**DOI:** 10.1186/s12912-025-03546-3

**Published:** 2025-07-12

**Authors:** Mayada Sobhy Zaki El-Bakry, Samia I. Hassan, Amina El-Nemer

**Affiliations:** Woman’s Health and Midwifery Nursing, Faculty of Nursing, Dakahlia Governorate, Mansoura University, Mansoura City, Egypt

**Keywords:** Cervical cancer, Attitude, Nursing staff, Prevention, Egypt

## Abstract

**Background:**

Cervical cancer is the third most commonly diagnosed cancer among women worldwide, while in Egypt, it is considered the 14th major contributor to female tumors.

**Aim of the study:**

To assess nursing staff attitude regarding prevention of cervical cancer.

**Methods:**

A descriptive cross-sectional study framework was executed in Mansoura University Hospital and six other buildings (psychiatry, burns, outpatient, special medicine, convalescence and radiation building), Mansoura city, Egypt, among 414 nursing staff who were chosen by using the convenience sampling technique. A structured interview questionnaire consisting of two parts was used to gather data from June 2024 and continued until the end of November 2024. Univariate analysis for descriptive data and bivariate analysis through the chi-square test were performed.

**Results:**

The current research revealed that 76.3% of the studied nursing staff had a positive attitude toward the prevention of cervical cancer, while 69.3% of them agreed that cervical neoplasm is a highly preventable disease, and 71.7% of them agreed that cervical neoplasm is curable if detected early. Also, there was a statistically significant positive association between educational level and attitude (*p* < 0.001) and a statistically significant positive association between marital status and attitude (*p* < 0.031). Also, there was a statistically significant positive association between vaccination against HPV and positive attitude (*p* < 0.001), while there was a statistically significant positive association between positive attitude and cervical cancer screening (*p* < 0.012).

**Conclusion:**

It is concluded that more than three-quarters of nursing staff demonstrated a positive attitude toward cervical cancer prevention, underscoring the need for continued educational interventions.

**Clinical trial number:**

This study was not registered as a clinical trial as it does not involve an interventional design.

**Supplementary Information:**

The online version contains supplementary material available at 10.1186/s12912-025-03546-3.

## Background

Cervical cancer is a malignant tumor of the cervical region. Also, cervical cancer is a significant general medical condition. It is one of the most well-known tumors affecting women of childbearing age between 20 and 45 years overall. Cervical cancer is a slow-growing malignancy primarily caused by HPV infection [1].

It frequently develops without any symptoms initially due to a long preinvasive period and has a bimodal peak at 35–39 years, 60–65 years [2]. It may take up to 15–20 years to develop without manifestations, and in later stages may result in bleeding between menses, bleeding after sexual intercourse, pelvic pain and/or dyspareunia, vaginal bleeding after menopause, foul odor, weight loss, dysuria and fatigue [3].

Also, cervical cancer is among the most frequent gynecological cancers in women. It is also one of the most prevalent female cancers that can be detected and treated in the precancerous stage. Sexually transmitted diseases (STDs), specifically HPV-16 and HPV-18, are the leading cause of cervical cancer. Other epidemiological risk factors are early sexual activity, teen pregnancy, family history and oral birth control [4].

Additionally, recent studies have demonstrated that HPV DNA testing plays a crucial role in triaging women with low-grade cytological abnormalities. This method has been shown to be more sensitive than the standard liquid-based cytology in the early detection of cervical intraepithelial neoplasia grade II or higher (CIN II+). Early diagnosis through HPV DNA testing enables timely intervention, which can significantly reduce the progression to cervical cancer. In addition to screening methods, preventive measures such as the HPV vaccine are essential in lowering the incidence of high-risk HPV infections and subsequently cervical cancer. Incorporating both HPV DNA testing and vaccination programs into cervical cancer prevention strategies is vital for improving women’s health outcomes [5].

In the setting of this study, conducted at Mansoura University Hospital, cervical cancer prevention services offered to women include routine Pap smear screening and limited availability of HPV DNA testing. The hospital also participates in public health initiatives that promote awareness of cervical cancer and encourage vaccination against HPV, although HPV vaccine coverage remains low among the target population. These services aim to improve early detection and prevention of cervical cancer among women attending the hospital [6].

According to the World Health Organization, around 530,000 women are affected by cervical cancer each year, with 275,000 dying as a result. It is said to be the 3rd most prevalent cause of cancer in women and the 2nd most common type of malignant progression in women in developing countries. Cervical cancer is the leading cause of death in women due to malignant progression in the majority of developing countries [7].

In Egypt, cervical carcinoma is the 14th major cause of female tumor of all ages, as well as the 11th most prevalent female tumor among women aged 15 to 44, with an estimated age-standardized incidence rate of 2.3 per 100,000 people annually. According to the World Health Organization in 2023, approximately 36.7 million Egyptian women are at risk of cervical carcinoma, with an incidence rate of 1320 cases/year. Cervical carcinoma is also the 12th major cause of cancer deaths among Egyptian women aged 15 to 44, with an age-standardized mortality rate of 1.5 per 100,000 people annually [8].

Additionally, cervical cancer is considered the 3rd most frequent type of cancer in women worldwide, following breast and colorectal cancer. Indeed, it is recognized as the disease with the greatest potential for prevention. According to research, HPV infection increases the risk of developing cervical cancer. Pap smear testing allows cervical lesions to be identified before they become cancerous, reducing the incidence of cervical cancer by 75 − 90% [9].

Nursing staff, especially maternity nurses, had a critical role in the prevention of cervical cancer since they are major players in the health care delivery system. Nursing staff should teach women of various ages to improve their knowledge and attitude. Nursing staff may be able to put their knowledge into practice and take on responsibility and accountability for women’s reproductive health, ultimately helping to enhance women’s reproductive health and reducing morbidity and mortality from cervical cancer [10].

### Significance of the study

Nursing staff play a critical role in cervical cancer prevention. Nursing staff interventions are beneficial for preventing, controlling, and treating early cervical cancer screening. Nursing staff must be knowledgeable and well equipped to be a good counsellor and effective communicators who provide education to women according to their status and education as well as play a role in the early detection and treatment of cervical cancer. So, it is critical to assess nursing staff knowledge and attitude about cervical cancer prevention [10].

The World Health Organization’s strategy for eliminating cervical cancer by 2030 includes ensuring that 90% of girls receive the full HPV vaccine by the age of 15, treating 90% of women diagnosed with cervical disease, and screening 70% of women with a high-performance test by the ages of 35 and 45. Achieving these goals could result in a 40% reduction in new cervical cancer cases and the prevention of 5 million deaths by 2050 [11].

## Methods

### Research design

A descriptive cross-sectional design was implemented. It is observational research that utilizes systematic and accurate descriptions of the facts and characteristics of a certain population or area of interest. Given the study’s purpose, a descriptive study is ideal for investigating nurses’ knowledge and attitudes toward cervical cancer prevention.

### Study setting

The study was carried out in all inpatient and outpatient units of the Mansoura University Hospital (MUH) and other buildings (convalescence building, psychiatric building, burns building, special medicine building, radiological building, outpatient clinics).

### Sample type

A convenient sample was used to recruit nursing staff who were available and willing to participate during the data collection period. The use of convenience sampling, which may limit the generalizability of the findings to the wider nursing staff.

### Sample size

Based on data from literature [12], considering the level of significance of 5%, and the power of the study of 80%, the sample size was calculated using the following formula:

n = (N × p (1-p))/((N-1)x(d^2/z^2) + p(1-p))

where n = sample size; N, studied total population; d = error percentage (= 0.05); p = prevalence or proportion of event of interest for the study; Zα/2 = 1.96 (for 5% level of significance). Therefore,

n = (2244 × 0.611 (1-0.611))/((2244-1)x(〖0.055〗^2/1.96〗^2) + 0.611(1-0.611)) = 413.6

Accordingly, the sample size required is 414.

### Study sample

The study utilized a convenient sample of 414 staff nurses who work at inpatient and outpatient departments (MUH 165 nurses, convalescence building 65 nurses, psychiatric building 28 nurses, burns building 46 nurses, special medicine building 51 nurses, radiological building 37 nurses, outpatient clinics 22 nurses).

### Inclusion criteria

Female practitioner nursing staff aged ≥ 20 years with at least 6 months of experience.

### Exclusion criteria

Nursing staff working in administrative roles, such as head nurses and supervisors.

Nursing staff aged < 20 years with less than 6 months of experience.

Male nursing staff.

Nursing staff who are currently on leave or unavailable during the study period.

Nursing staff who decline to participate or do not provide informed consent.

### Tool of data collection

Data was collected using one tool:

#### Tool (I): A structured interview questionnaire

**The tool was developed by the researcher after reviewing the related national & global literature – it is composed of two parts.**

**Part 1: Nursing staff General Characteristics**, such as age, residence, educational level, years of working, health care worker qualification, department, building,……etc. Also, **Self-reported experience regarding cervical cancer**,** its vaccination and screening.**

**Part 2**: Nurse’s Attitude regarding prevention of cervical cancer: It was developed by the researcher after reviewing the related national & global literature [13]. It consisted of 24 statements related to attitude regarding cervical cancer prevention, such as: Cervical cancer is a highly preventable disease, cervical cancer is curable if detected early, screening is not expensive, awareness campaigns have a significant role in the prevention of cervical cancer, etc.

“Positive attitude was defined as a nurse’s favorable beliefs and emotions toward cervical cancer prevention and early screening, but negative attitude refers to reluctance, misconceptions, or resistance toward cervical cancer screening practices.”

**The full questionnaire used in this study is available in Supplementary File** 1.

### Scoring system

A three-point Likert scale was used (1 disagree, 2 neutral, 3 agree). The respondent evaluated each response (1:3). The replies were counted to get a total score, and then the mean score was determined. Those scoring above the mean score (≥ 56%) had a positive attitude towards cervical cancer prevention, whereas those scoring below the mean score (< 56%) had a negative attitude. The 56% cutoff was adapted from a previous study with a similar attitude scale and scoring method [14].

### Tool validity

Before using the tools, a panel of five experts in Women’s Health and Midwifery Nursing reviewed their content validity to ensure that the questions were consistently conveyed and carried the anticipated meaning that they were prepared for. Modifications were made (e.g., simplifying the meaning of some questions and rearranging the sequence of some questions), and the final form was used for data collection. **The tool was developed by the researcher after reviewing the related national & global literature.**

### Tool reliability

The questionnaire was pilot-tested and indicated its reliability (Cronbach’s alpha = 0.879) which indicated high reliability.

### Ethical considerations

The Research Ethics Committee at Mansoura University’s Faculty of Nursing granted ethical permission (number 452). Nurses were informed that their participation in the trial was entirely voluntary. They (Research Ethics Committee) also stated that each nurse has the freedom to withdraw from research at any time with no penalty. After describing the goal of the study, all the nurses studied gave their informed written consent. Nurses were assured of the confidentiality of the collected data, and the results were used as part of the required study for a master’s degree, as well as future publications and education.

### Study process

The process was divided into two phases: the Preparatory phase and the operating phase.

### Preparatory phase

During this phase, the researcher attained official approval from the director of the previous setting. After that, followed by an assessment of relevant national and international literature, data collection instruments were developed in Arabic. Before collecting the actual study sample, a pilot study was carried out on 10% of the sample size and excluded from the sample.

### Pilot study

The pilot study was conducted on 10% of the entire sample (42 nurses) to evaluate the tools’ simplicity, adaptability, clarity, and applicability. Based on the findings of the pilot study, essential modifications were made, such as reducing the meaning of some phrases, integrating some questions to improve clarity and relevance and the pilot study was omitted from the sample. This stage finished in May 2024.

### Operating phase

#### Data collection phase

- The real fieldwork for data collecting began in early June 2024 and continued until the end of November 2024. The researcher attended two days per week (Saturday and Wednesday) (not hot days) at the time when the nurse was available after performing her duties until the calculated sample was obtained between 1 and 5 p.m. (average number of 10 nursing staff/day). In the previously mentioned setting to complete data collection using the questionnaire.
- The researcher presented herself to the nursing staff, explained the study’s purpose, ensured that the nurses met the study’s criteria, and acquired written consent from nursing staff to participate in the study after confirming data confidentiality and that they have the right to withdraw from the study at any time.
- The researcher gave each nurse the questionnaire to get information about their general characteristics and attitude toward cervical cancer prevention.
- During this process, the nursing staff read each question on the form and documented her answers.
- The researcher attended the previously indicated location until the sample size was met.

#### Data analysis phase

The data obtained was saved, organized, categorized, transferred to specially prepared formats, and statistically analyzed. All statistical analyses were carried out using SPSS version 22. The continuous data was normally distributed and offered as Mean ± Standard Deviation (SD). Categorical data was presented numerically and as percentages. To compare variables using categorical data, the chi-square test (or Fisher’s exact test when applicable) was utilized. The co-efficient correlation test was utilized to investigate association between 2 variables with continuous data. The internal consistency (reliability) test for the questionnaires used in the study was calculated. Statistical significance was set at *p* < 0.05.

## Results

Table 1. Demonstrates that the average age of the studied nursing staff was **(27.99 ± 5.87)** years, **(46.9%)** of studied nurses live in urban areas, **(56.5%)** of them were married, **(63%)** had Nursing Institute, average years of experience of the studied nursing staff were **(6.31 ± 5.41)** years, **(39.9%)** of them were from MUH and **(5.3%)** of them were from outpatient clinics. As well as **(15.7%)** of them were in the obstetric department.

**Table 1:** Distribution of the studied nursing staff according to their general characteristics (*n* = 414)

Variable	*N*	%
**Age/years**		
20–24	140	33.8
25–29	149	**36.0**
30–34	58	14.0
35–39	53	12.8
40–44	14	3.4
**Mean ± SD (Range)**	**27.99 ± 5.87 (20–45)**	
**Residence**		
Urban	194	**46.9**
Rural	220	53.1
**Marital status**		
Single	143	34.5
Married	234	**56.5**
Divorced	19	4.6
Widow	18	4.4
**Educational level**		
Diploma degree	62	15.0
Nursing Institute	261	**63.0**
Bachelor’s degree in nursing	79	19.1
Postgraduate	12	2.9
**Years of experience**		
≥ 6 months- < 5 years	203	**49.0**
5- < 10 years	118	28.5
10- < 15 years	44	10.7
≥ 15 years	49	11.8
**Mean ± SD (Range)**	6.31 ± 5.41 (1–25)	
**Hospital buildings**		
Outpatient clinics	22	**5.3**
Psychiatric building	28	6.8
Nuclear medicine building	37	8.9
Burns building	46	11.1
Special medicine building	51	12.3
Convalescence building	65	15.7
Mansoura University Hospital (MUH)	165	**39.9**
**Departments**		
Obstetric departments	65	**15.7**
Other departments	349	**84.3**

Table 2. Demonstrates that the studied nursing staff agree that cervical cancer is a highly preventable disease, cervical cancer is curable if detected early, cervical cancer can be detected in the earliest stages, screening can detect cervical cancer early, screening helps in the prevention of cervical carcinoma, discuss pap smear with their patients and others before, pap smear is more accurate so, they encourage it compared to other methods, pap test helps to decrease maternal morbidity and mortality and cervical cancer is not a serious health problem, so screening is just a burden 69.3%, 71.7%, 66.7%, 51.7%, 59.2%, 43.5%, 51%, 54.3% and 44.7% respectively.

**Table 2:** Distribution of the studied nursing staff according to their attitude regarding prevention of cervical cancer (*n* = 414)

Item	Disagree		Neutral		Agree	
	*N*	%	*N*	%	*N*	%
1. Cervical cancer is a highly preventable disease.	65	15.7	62	15.0	287	**69.3**
2.Cervical cancer is curable if detected early.	52	12.6	65	15.7	297	**71.7**
3.Cervical cancer can be detected in the earliest stages.	76	18.4	62	15.0	276	**66.7**
4.Avoiding early marriage before 18 years old can prevent cervical cancer.	85	20.5	108	26.1	221	**53.4**
5. Maintaining sexual hygiene can prevent cervical cancer.	91	22.0	243	**58.7**	80	19.3
6.Avoiding multiple sexual partners can prevent cervical cancer.	79	19.1	88	21.3	247	**59.7**
7.Balanced nutrition helps in the prevention of cervical cancer.	74	17.9	129	31.2	211	**51.0**
8. Strong immunity has an important role in the prevention of cervical cancer.	226	**54.6**	113	27.3	75	18.1
9. Awareness campaigns have a significant role in the prevention of cervical cancer.	84	20.3	257	**62.1**	73	17.6
10.Human Papilloma Virus (HPV)vaccine is not effective in the prevention of cervical cancer. ^®^	165	**39.9**	96	23.2	153	37.0
11.It is important to vaccinate your children against HPV.	229	**55.3**	72	17.4	113	27.3
12.Screening can detect cervical cancer early.	127	30.7	73	17.6	214	**51.7**
13.Screening helps in the prevention of cervical carcinoma.	112	27.1	57	13.8	245	**59.2**
14. Screening for cervical cancer is not expensive. ^®^	157	**37.9**	123	29.7	134	32.4
15. Always advise the patients for screening methods.	188	**45.4**	64	15.5	162	39.1
16. Cervical cancer can be diagnosed easily.	177	**42.8**	77	18.6	160	38.6
17.A woman can easily accept different methods of diagnosis. ^®^	168	**40.6**	125	30.2	121	29.2
18.If you were told that VIA test is simple, painless, costless and good for early detection of cervical cancer, would you like to, have it?	216	**52.2**	118	28.5	80	19.3
19. Discuss pap smear with your patients and others before.	118	28.5	116	28.0	180	**43.5**
20.From your point of view, the pap smear is more accurate so, you encourage it compared to other methods.	113	27.3	90	21.7	211	**51.0**
21.Pap test helps to decrease maternal morbidity and mortality.	112	27.1	77	18.6	225	**54.3**
22.Cervical cancer is not a serious health problem, so screening is just a burden. ^®^	179	43.2	50	12.1	185	**44.7**
23.Participating in a training program is very important for cervical cancer prevention.	114	27.5	205	**49.5**	95	22.9
24.Nursing job plays a role in cervical cancer prevention.	114	27.5	230	**55.6**	70	16.9
Total score	**50.64 ± 9.64**					

R = Reverse Coding

Additionally, the studied nursing staff neutral that maintaining sexual hygiene can prevent cervical cancer, awareness campaigns have an important role in the prevention of cervical cancer, participating in a training program is very important for cervical cancer prevention and nursing job plays a role in cervical cancer prevention 58.7%, 62.1%, 49.5% and 55.6% respectively. Also, the studied nursing staff disagree that HPV vaccine is not effective in the prevention of cervical cancer, it is important to vaccinate your children against HPV, screening for cervical cancer is not expensive, they always advise the patients to screen for cervical cancer and woman can easily accept different methods of diagnosis 39.9%, 55.3%, 37.9%, 45.4% and 40.6% respectively.

**Fig. 1:**
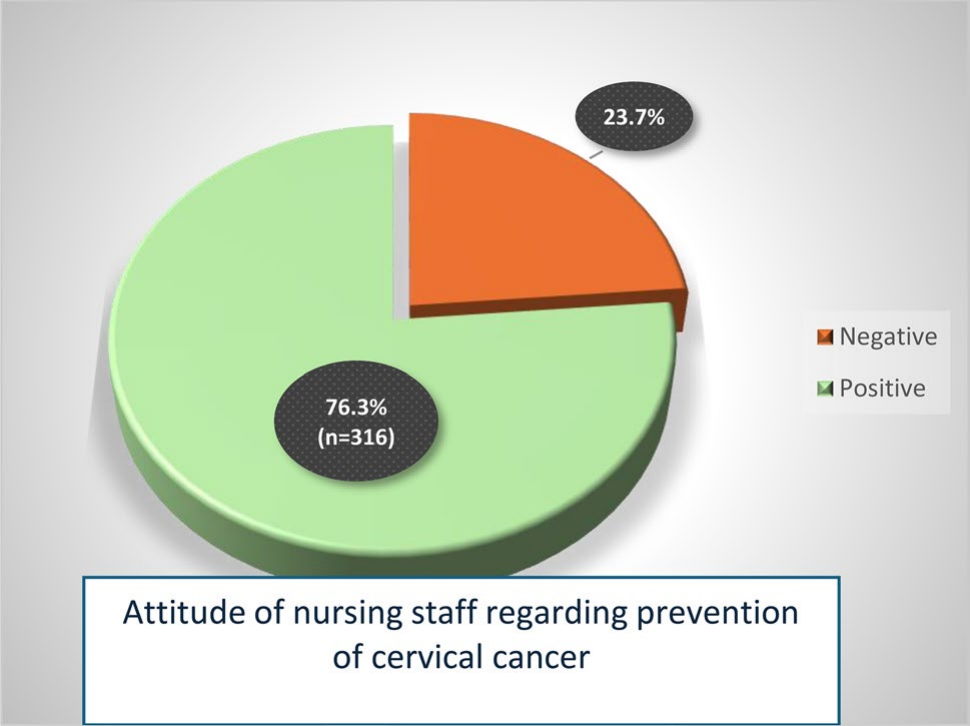
Total attitude score of the studied nursing staff regarding prevention of cervical cancer (***n*** **= 414**)

Figure 1. Illustrates that **(76.3%)** of the studied nursing staff had a positive attitude regarding prevention of cervical cancer and **(23.7%)** had a negative attitude regarding prevention of cervical cancer.

Table 3. Represents that there was a statistically significant association between knowledge of the studied nursing staff regarding cervical cancer and its prevention and marital status (*P* = 0.031) in which 79.1% of the studied nursing staff with positive attitude was married. Also, there was a statistically significant association between positive attitude and studied nursing staff level of education (*P* < 0.001) which 100% of them postgraduate.

**Table 3:** Relation between general characteristics of the studied nursing staff and their attitude toward cervical malignancy and its prevention (***n*** **= 414**)

Variable	Negative attitude (*n* = 98)		Positive attitude (*n* = 316)		DF	Significance tests	
	*N*	%	*N*	%		X^2^	(*P*)
**Age/years**							
20–24	33	23.6	107	76.4	4	8.079	(0.089)
25–29	26	17.4	123	82.6			
30–34	18	31.0	40	69.0			
35–39	18	34.0	35	66.0			
40–44	3	21.4	11	78.6			
**Residence**							
Urban	50	25.8	144	74.2	1	0.892	(0.345)
Rural	48	21.8	172	78.2			
**Marital status**							
Single	33	23.1	110	76.9	3	8.866	(0.031) ^*^
Married	49	20.9	185	**79.1**			
Divorced	8	42.1	11	57.9			
Widow	8	44.4	10	55.6			
**Educational level**							
Diploma degree	50	80.6	12	19.4	3	32.295	(< 0.001) ^**^
Nursing Institute	37	14.2	224	85.8			
Bachelor’s degree in nursing	11	13.9	68	86.1			
Postgraduate	0	0.0	12	**100.0**			
**Years of experience**							
≥ 6 months-< 5 years	5	26.6	149	73.4	3	3.227	(0.358)
5- < 10 years	26	22.0	92	78.0			
10-< 15 years	7	15.9	37	84.1			
≥ 15 years	11	22.4	38	77.6			
**Hospital building**							
Psychiatric building	18	64.3	10	35.7	6	34.047	(< 0.001) ^**^
Special medicine building	11	21.6	40	78.4			
Outpatient clinics	8	36.4	14	63.6			
Nuclear medicine building	9	24.3	28	75.7			
Convalescence building	12	18.5	53	81.5			
Burns building	13	28.3	33	71.7			
MUH	27	16.4	138	**83.6**			
**Department**							
Obstetric department	11	16.9	54	83.1	1	1.943	)0.163)
Others department	87	24.9	262	75.1			

Note: DF: Degree of Freedom χ2: Chi -square test *(P) Significant at *P* ≤ 0.05 ** significant at *P* ≤ 0.001

Table 4. Represents that, there was a statistically significant association between attitude of the studied nursing staff regarding cervical cancer and its prevention and vaccinated against HPV (*P* < 0.001) in which 96.6% of the studied nursing staff who had positive attitude vaccinated against HPV. Also, there was a statistically significant association between positive attitude and cervical cancer screening (*P* = 0.012) in which 96.3% of them performed cervical cancer screening.

**Table 4:** Relationship between studied nursing staff self-reported experience and attitude regarding cervical cancer and its prevention (***n*** **= 414**)

Variable	Negative attitude (*n* = 98)		Positive attitude (*n* = 316)		DF	Significance tests	
	*N*	%	*N*	%		X^2^	(*P*)
**Previous courses**							
No	96	24.1	303	75.9	1	2.491	(0.115)
Yes	2	13.3	13	86.7			
**Vaccination against HPV**							
No	95	29.2	230	70.8	1	25.859	(< 0.001) ^**^
Yes	3	3.4	86	**96.6**			
**Perform cervical cancer screening**							
No	97	25.1	290	74.9	1	6.374	(0.012) ^*^
Yes	1	3.7	26	**96.3**			

Note: DF: Degree of Freedom χ2: Chi -square test *(P) Significant at *P* ≤ 0.05 ** significant at *P* ≤ 0.001

## Discussion

The current study aims to assess nursing staff attitude toward prevention of cervical cancer. This goal was accomplished by the current research results, which revealed that more than three-quarters of nursing staff had a positive attitude toward cervical cancer prevention. This result agreed with [12], who studied knowledge, attitude, barriers and practices of cervical cancer prevention among nurses in selected hospitals in the Eastern Cape Province, South Africa that found more than three-quarters of the studied nursing staff had a positive attitude regarding prevention of cervical cancer. The reason behind this could be because nurses who took part in the 2020 national cervical cancer screening campaign, which emphasized the significance of screening behavior and its role in cancer prevention, had a more positive attitude.

On the other hand, this study contradicted [15], who assessed female healthcare providers’ knowledge, attitude, and practice towards cervical cancer screening and associated factors in public hospitals of Northwest Ethiopia that reported only one third of the nursing staff had positive attitude regarding prevention of cervical cancer. The probable cause of the disparity could be differences between health care providers as well as differences in values and beliefs across the study groups and locations in general between the present and prior studies.

The study results showed that more than two-thirds of the studied nursing staff agreed that Cervical cancer is a highly preventable disease. This result in agreement with [16], who studied knowledge, attitude, and practice of cervical cancer screening among the healthcare workers of Western Region, Nepal that found more than two-thirds of the studied nursing staff agreed that Cervical cancer is a highly preventable disease. This might be due to Nepal issued National Guidelines for cervical Cancer Screening and Prevention.

The present study results found that exceeding fifty of the studied nursing staff agreed that screening can detect cervical neoplasm early and helps in the prevention of cervical neoplasm. These results supported by [17], who assessed knowledge, attitude, and practice of healthcare workers in Ekiti State, Nigeria on prevention of cervical cancer revealed that exceeding fifty of the studied nursing staff agreed that screening can detect cervical cancer early and helps in the prevention of cervical neoplasm. This implies the importance of cervical cancer screening programs and to take advantage of nurse’s favorable attitude.

The research findings demonstrated that exceeding one third of the studied nursing staff agreed that HPV vaccine is effective in the prevention of cervical neoplasm. This result consistent with [18], who studied knowledge, attitude and practice of cervical cancer: prevention, screening and management among nurses at tertiary care hospitals of Faisalabad found that more than one third of the studied nursing staff agreed that HPV vaccine is effective in the prevention of cervical neoplasm. This may be due to limited training and cultural sensitivity around discussing sexually transmitted infections. Enhancing educational programs could help improve nurses’ understanding and support for HPV vaccination.

The present research results demonstrated that less than half of the investigated nursing staff disagreed that they advise their women about screening. These findings are consistent with [19], who assessed challenges regarding the implementation of cervical cancer screening guidelines in Limpopo province, South Africa found that less than half of the studied nursing staff disagreed that they advise their patients to screen for cervical cancer. This may be due to lack of training, time constraints, or unclear institutional guidelines, indicating a need for stronger support and clearer protocols for cervical cancer screening.

The current study results revealed that about half of the studied nursing staff neutrally believe that participating in a training program is particularly important for cervical cancer prevention. Similar trends were reported in [20], who studied knowledge, attitude, and practice of healthcare workers in Ekiti State, Nigeria on prevention of cervical cancer reported that about half of the studied nursing staff neutrally believe that participating in a training program is particularly important for cervical cancer prevention. It indicate insufficient exposure to training opportunities or limited understanding of how such programs can improve clinical practice. This points to a need for more engaging and accessible training initiatives.

The current study results found that there was a statistically significant association between attitude and marital status. This result in agreement with by [21], who assessed cervical cancer screening risk perception, uptake, and associated factors among female primary healthcare workers in Edo State, Nigeria reported that there was a statistically significant association between attitude and marital status. This may be due to married nursing staff prioritizing work life balance, influencing and shaping attitude toward cervical cancer.

Regarding the relation between self-reported experience of cervical neoplasm screening and prevention of the studied nurses and their attitude regarding cervical cancer & its prevention, the current study found that there was a statistically significant association between attitude and performance of cervical cancer screening. This result supported by [22], demonstrated that there was a statistically significant association between positive attitude and performing cervical cancer screening. It may be explained by the idea that nurses with positive attitudes are more inclined to participate in and promote cervical cancer screening, as attitudes strongly influence behavior.

On the other hand, this study contradicted with [23], who assessed knowledge, attitude, and practice toward cervical cancer screening among female nurses in Ishaka Western Uganda reported that there was no statistically significant association between attitude and performing cervical cancer screening. This may be due to differences in age group, cultural beliefs and traditions. This difference may be justified by variations in participants’ age, cultural norms, and healthcare practices between settings, which can influence both attitudes and behaviors.

The present research result revealed that there was a statistically significant association between positive attitude and vaccinated against HPV. Similar trends were reported in [24], who studied difference between awareness and uptake of cervical cancer screening among health care workers and [25], who knowledge, attitude, and practice on screening toward cervical cancer among nursing staff in India reported that there was a statistically significant association between positive attitude and vaccinated against HPV. This may be explained by the fact that nurses with positive attitudes are more likely to accept and seek HPV vaccination, believing in its preventive value.

## Conclusion

It is concluded that exceeding three quarters of nursing staff demonstrated a positive attitude concerning cervical cancer & its prevention. Moreover, exceeding fifty of them agreed that screening help in the prevention of cervical cancer and it can detect cervical cancer early however, nearly half of them reported not advising their patients for screening methods because majority of them from the other departments rather than obstetric departments, so, it is beyond their scope of interest in a non-obstetrics department and not related to their work specialty.

### Recommendations

- Establish national community-based awareness campaigns about the presidential initiative for screening and early identification of cervical neoplasm through social & mass media.
- Developing cervical cancer screening training program for nurses to be able to support and educate women about cervical cancer prevention and screening methods.

### Further studies

- Evaluating the effectiveness of awareness campaigns regarding cervical cancer prevention and screening methods on nursing staff acceptance on performing screening.
- Explore the underlying factors influencing nursing staff decisions to participate in cervical cancer screening.
- Replication of the present study on different geographical areas in Egypt.

### Study limitations

This study employed a convenience sampling technique, which may introduce selection bias and limit the external validity of the findings. As the participants were not randomly selected and were drawn from a specific setting, caution should be exercised when generalizing the results to broader populations. Future research using random or stratified sampling across diverse settings is recommended to improve generalizability and strengthen methodological rigor.

Data collection was conducted during specific days and working hours, which may have excluded nurses working night shifts or weekends. This timing bias could limit the representativeness of the sample and affect the generalizability of the study findings.

### Implications for nursing practice

The findings of this study highlight the importance of enhancing nursing staff awareness and attitudes toward cervical cancer prevention. Positive attitudes among nursing staff can directly influence the quality of patient education and screening practices. Therefore, incorporating continuous education and training programs focused on cervical cancer, HPV vaccination, and early detection into nursing practice is essential. This can empower nursing staff to play a proactive role in community health promotion and reduce cervical cancer incidence through effective prevention strategies.

## Electronic supplementary material

Below is the link to the electronic supplementary material.

Supplementary Material 1

## Data Availability

The datasets used and analyzed during the current study are available.from the corresponding author on reasonable request.

## Abbreviations

CIN: Cervical Intraepithelial Neoplasia
HPV: Human Papillomavirus
SD: Standard Deviation
SPSS: Statistical Package for Social Sciences
STDS: Sexually Transmitted Diseases
WHO: World Health Organization
MUH: Mansoura University Hospital
