# Supplementary material for: Assessment of nursing staff attitude regarding prevention of cervical cancer
Source: BMC Nurs. 2025 Jul 12;24:910. doi: 10.1186/s12912-025-03546-3 (PMC12254958; doi:10.1186/s12912-025-03546-3)
Supplement: Supplementary file 1 — Supplementary Material 1 [file 12912_2025_3546_MOESM1_ESM.docx]

**Data Collection to assess nursing staff knowledge and attitude regarding prevention of cervical cancer**

**Part1: 1-** **Nursing staff General characteristics**

1-Hospital building :………………………………

2-Department:………………………..

3-Age:…………………years

A-20: < 25 years ( ) B-25 : < 30 years ( )

C-30: < 35 years ( ) D-35 : < 40 years ( )

E-More than 40 years ( )

4-Residence:

A-Urban( ) B-Rural ( )

5-Marital status:

A- Single( ) B-Married( )

C-Divorced( ) D-Widow( )

6-Level of education:

A-Diploma of nursing( ) B- Technical institute of nursing ( )

C- Bachelor of nursing ( ) D-Postgraduate( )

7- Years of experience…………………years

A-6 months: < 5 years ( ) B-5 : < 10 years ( )

C-10: < 15 years ( ) D- ≥15 years ( )

**2- Self-reported experience regarding cervical cancer, its vaccination and screening**

8-Do you heard about cervical cancer?

A-Yes ( ) B-No ( )

9-If yes: what are your sources of information about cervical cancer? (you can choose more than one answer)

A-Relatives and/or friends( ) B-Books( )

C-Health care provider ( ) D-Mass media( )

E-Other( )

10-Did you take any training courses regarding cervical cancer prevention?

A-Yes ( ) B-No( )

5-Have you heard of the HPV vaccine?

A-Yes( ) B- No( )

11-Are you vaccinated against HPV?

A-Yes ( ) B-No ( )

12-If no What are reasons for not vaccinated? (you can choose more than one choice)

A-Vaccine is too expensive( ) B-Vaccine is not available ( )

C-Vaccine is not effective ( ) D-Fear from complications or side effects ( )

13-Have you ever heard about cervical cancer screening?

A-Yes( ) B-No( )

14- If yes: What are the sources of information about cervical cancer screening? (you can choose more than one choice)

A-Relatives and/or friends( ) B-Health care provider( )

C-Mass media( ) D-Other( )

15-Do you have cervical cancer screening?

A-Yes( ) B-No( )

16- what are the reasons for not initiating cervical cancer screening? (you can choose more than one choice)

A-No symptoms, no need( ) B-Feel of shyness of having gynecological examination ( )

C-Fear from result of screening( ) D-Fear of technique of screening( )

E-No time to go for it ( ) F-Screening is expensive ( )

G-Other ( )

Part2:

**Part2: Attitude of nursing staff** **about prevention of cervical cancer**

| **Item** | **Disagree**  **(1)** | **Neutral**  **(2)** | **Agree**  **(3)** |
| --- | --- | --- | --- |
| 1. Cervical cancer is a highly preventable disease. |  |  |  |
| 1. Cervical cancer is curable if detected early. |  |  |  |
| 1. Cervical cancer can be detected in the earliest stages. |  |  |  |
| 1. Avoiding early marriage before age 18 years can prevent cervical cancer. |  |  |  |
| 1. Maintaining sexual hygiene can prevent cervical cancer. |  |  |  |
| 1. Avoiding multiple sexual partners can prevent cervical cancer. |  |  |  |
| 1. Balanced nutrition helps in the prevention of cervical cancer. |  |  |  |
| 1. Strong immunity has an important role in the prevention of cervical cancer. |  |  |  |
| 1. Awareness campaigns have an important role in the prevention of cervical cancer. |  |  |  |
| 1. HPV vaccine is not effective in the prevention of cervical cancer. |  |  |  |
| 1. It is important to vaccinate your children against HPV. |  |  |  |
| 1. Screening can detect cervical cancer early. |  |  |  |
| 1. Screening helps in the prevention of cervical carcinoma. |  |  |  |
| 1. Screening for cervical cancer is not expensive. |  |  |  |
| 1. Always advise the patients to screen for cervical cancer. |  |  |  |
| 1. Cervical cancer can be diagnosed easily. |  |  |  |
| 1. A woman can easily accept different methods of diagnosis. |  |  |  |
| 1. If you were told that VIA test is simple, painless, costless and good for early detection of cervical cancer, would you like to, have it? |  |  |  |
| 1. Discuss pap smear with your patients and others before. |  |  |  |
| 1. From your point of view pap smear is more accurate so, you encourage it compared to other methods. |  |  |  |
| 1. Pap test helps to decrease maternal morbidity and mortality. |  |  |  |
| 1. Cervical cancer is not a serious health problem, so screening is just a burden. |  |  |  |
| 1. Participating in a training program is very important for cervical cancer prevention. |  |  |  |
| 1. Nursing job plays a role in cervical cancer prevention. |  |  |  |
